# Zinc Modulates Endotoxin-Induced Human Macrophage Inflammation through ZIP8 Induction and C/EBPβ Inhibition

**DOI:** 10.1371/journal.pone.0169531

**Published:** 2017-01-05

**Authors:** Charlie J. Pyle, Saife Akhter, ShengYing Bao, Claire E. Dodd, Larry S. Schlesinger, Daren L. Knoell

**Affiliations:** 1 Center for Microbial Interface Biology, The Ohio State University, Columbus, Ohio, United States of America; 2 Dorothy M. Davis Heart and Lung Research Institute, The Ohio State University, Columbus, Ohio, United States of America; 3 College of Pharmacy, The University of Nebraska Medical Center College of Pharmacy, Omaha, Nebraska United States of America; 4 Department of Microbiology, The Ohio State University, Columbus, Ohio, United States of America; 5 Department of Microbial Infection and Immunity, The Ohio State University, Columbus, Ohio, United States of America; Friedrich-Alexander-Universitat Erlangen, GERMANY

## Abstract

Two vital functions of the innate immune system are to initiate inflammation and redistribute micronutrients in favor of the host. Zinc is an essential micronutrient used in host defense. The zinc importer ZIP8 is uniquely induced through stimulation of the NF-κB pathway by LPS in monocytes and functions to regulate inflammation in a zinc-dependent manner. Herein we determined the impact of zinc metabolism following LPS-induced inflammation in human macrophages. We observed that ZIP8 is constitutively expressed in resting macrophages and strikingly elevated following LPS exposure, a response that is unique compared to the 13 other known zinc import proteins. During LPS exposure, extracellular zinc concentrations within the physiological range markedly reduced IL-10 mRNA expression and protein release but increased mRNA expression of TNFα, IL-8, and IL-6. ZIP8 knockdown inhibited LPS-driven cellular accumulation of zinc and prevented zinc-dependent reduction of IL-10 release. Further, zinc supplementation reduced nuclear localization and activity of C/EBPβ, a transcription factor known to drive IL-10 expression. These studies demonstrate for the first time that zinc regulates LPS-mediated immune activation of human macrophages in a ZIP8-dependent manner, reducing IL-10. Based on these findings we predict that macrophage zinc metabolism is important in host defense against pathogens.

## Introduction

Micronutrient metabolism plays a critical role in innate immune defense against microbial infection. Macrophages exploit the biochemical characteristics of transition metals in part by manipulating their uptake and trafficking following pathogen recognition. Cation re-distribution from extracellular and intracellular compartments into the cell cytosol in response to infection benefits the host in a number of important ways. It inhibits pathogen growth and survival through deprivation of indispensable micronutrients, generates host protective Fenton-reaction-dependent reactive oxygen species and affords nonspecific inhibition of bacterial protein binding [1–3]. Importantly, internalized micronutrients also help orchestrate vital signaling pathways [3–6]. Zinc is an essential micronutrient utilized in host defense. Inadequate zinc nutrition reduces innate immune competence, thereby increasing susceptibility to infectious disease [7]. Human zinc metabolism, which is primarily controlled by fourteen ZIP (Zrt/Irt-like protein) zinc import proteins and ten cytosolic zinc export proteins (ZnTs), is altered by microbial-initiated activation of innate immune cells [8, 9].

Lipopolysaccharide (LPS) stimulates human macrophage gene transcription following Toll-like receptor 4 (TLR4) binding and sequential activation of intracellular biochemical signaling cascades. The resulting nuclear localization and activation of a number of transcriptional co-activators and transcription factors including but not limited to nuclear factor kappa-light-chain-enhancer of activated B cells (NF-κB) and CCAAT/enhancer binding protein beta (C/EBPβ) largely determines the inflammatory response to infection [10, 11]. Monocytes respond to recognition of LPS by increasing transcription of the zinc transporter SLC39A8 (ZIP8) [12]. ZIP8 is induced through the canonical NF-κB pathway following LPS exposure resulting in translocation of ZIP8 protein to the plasma membrane and intracellular vesicles, and zinc import into the cytoplasm. The newly formed zinc pool in turn reduces further NF-κB activity through inhibition of I kappa-B kinase (IKK) activity [4, 13]. NF-κB is responsible in part for production of pro-inflammatory cytokines and chemokines that include but are not limited to tumor necrosis factor alpha (TNFα), interleukin eight (IL-8) and interleukin six (IL-6) [10]. LPS stimulation of human macrophages also induces the immune modulatory cytokine interleukin ten (IL-10) [14–16]. IL-10 production by LPS-stimulated macrophages occurs following phosphorylation of the IKK complex and mitogen-activated protein kinases (MAPKs), that regulate activation of transcription factors including cAMP response element-binding protein (CREB), activator protein one (AP-1), C/EBPδ, C/EBPβ and NF-κB subunit p50 (p50). Concurrent activation of the transcriptional co-activators CREB-binding protein (CBP) and p300 also enhance the IL-10 response. [17–21].

Macrophages differ significantly from monocytes in their phenotype and function. The metabolic pathways responsible for zinc trafficking during macrophage host defense have only begun to be explored [3, 22]. In response to microbes, macrophages produce both pro-inflammatory cytokines and IL-10 in order to coordinate a localized and balanced response aimed at efficiently eliminating infection while minimizing damage to surrounding tissue. IL-10 production by human macrophages in response to infection is essential for regulating immune responses through both autocrine and paracrine feedback mechanisms [17]. Importantly, IL-10 stimulation of murine and human macrophages significantly reduces production of pro-inflammatory cytokines [15, 16, 23, 24].

We hypothesized that ZIP8-mediated transport of extracellular zinc into human macrophages has the capacity to modulate the balance of pro- and anti-inflammatory cytokines produced in response to LPS. Consequently, deficits in zinc metabolism would disrupt macrophage function through modulation of zinc-dependent signaling pathways that alter host defense. In this study we determined that ZIP8 is constitutively expressed in macrophages and substantially induced following LPS exposure. Most striking, ZIP8-mediated zinc uptake within hours of LPS stimulation resulted in reduced IL-10 production and an increase in the pro-inflammatory cytokine response. Furthermore, nuclear accumulation and activation of the IL-10 inducing transcription factor C/EBPβ is reduced by zinc supplementation of LPS-exposed macrophages.

## Materials and Methods

### Reagents

TRIzol, RPMI 1640 with L-glutamine, and DPBS were purchased from Invitrogen (Carlsbad, CA). Zinc Sulfate heptahydrate, LPS L-4516 from *E*.*coli* and BSA were purchased from Sigma-Aldrich (St. Louis, MO). Ficoll-Paque and TMB substrate were purchased from GE Healthcare (Little Chalfont, UK). Rabbit polyclonal antiserum anti-peptide to amino acid residues 225–243 of human ZIP8 (1:1000) was purchased from Covance (Princeton, NJ). Mouse anti-human monoclonal β-Actin (#69101) (1:10,000) antibody was purchased from MP Biomedicals (Santa Ana, CA). Rabbit anti-human monoclonal Lamin B1 (#377001) (1:1000) antibody was purchased from Santa Cruz Biotechnology (Santa Cruz, CA). Rabbit anti-human monoclonal C/EBPβ (#1479–1) (1:1000) antibody was purchased from Epitomics (Burlingame, CA). Rabbit anti-human monoclonal NF-κB1 p105/p50 (#12540) (1:2000), polyclonal ERK (#4695) (1:1000), polyclonal p-ERK (#9101) (1:1000), polyclonal p38 (#9212) (1:2000), monoclonal p-p38 (#9215) (1:2000) and polyclonal p-C/EBPβ (#3084) (1:1000) antibodies were purchased from Cell Signaling Technology (Danvers, MA). C/EBPβ TransAM ELISA kit was purchased from Active Motif (Carlsbad, CA). ZIP8 epitope specific, 21mer small interfering RNA (siRNA) (target sequence TAGGACTTAGGAAATAAATAA) and scramble control siRNA were purchased from QIAGEN (Hilden, DEU).

### Human monocyte-derived-macrophage zinc supplementation model

Blood was collected from healthy human volunteers under a protocol approved by the Ohio State University’s Office of Responsible Research Practices (IRB), with written donor consent. Human peripheral blood mononuclear cells (PBMCs) were then isolated from heparinized blood on a Ficoll-Paque cushion (GE Healthcare) as previously described [25]. Differentiation of monocytes into monocyte-derived macrophages (MDMs) was accomplished by incubation of PBMCs (2x10^6^/mL) within Teflon wells (Savillex) at 37°C with 5% CO_2_ over a period of five days in RPMI 1640 medium containing 20% autologous serum, which contains < 3 μM zinc. MDMs in the PBMCs were placed in monolayer culture (99% pure), washed and repleted with RPMI containing 2% autologous serum or 10% autologous serum, then repleted with or without 10 μM, 18 μM or 40 μM Zinc Sulfate, with or without LPS 100 ng/mL.

### Transfection of MDMs

PBMCs were transfected with 50 nM ZIP8 or control siRNA using the Amaxa Nulceofector (Lonza) [26] as directed by the manufacturer. Following transfection PBMCs were seeded onto tissue culture plates and incubated in RPMI-1640 with 10% autologous serum for 2 h at 37°C in 5% CO_2_. Lymphocytes were removed by washing with warm RPMI-1640. Adhered, transfected MDMs were repleted with RPMI-1640 containing 20% autologous serum and incubated overnight at 37°C in 5% CO_2_ to allow recovery before further treatment.

### Cytokine assay

Cell free supernatants were collected from MDM monolayers at 6 and 24 h and used to measure IL-6, IL-8, IL-10 and TNFα by ELISA (R&D Systems).

### Protein lysate preparation and Western blot

MDM monolayers were lysed with TN1 buffer to generate whole cell lysate, then incubated at 4°C for 10 min. Lysates were centrifuged at 17,949 × *g* at 4°C to remove cell debris. To generate nuclear isolates, MDM monolayers were lysed and processed with NE-PER nuclear and cytoplasmic extraction reagents (ThermoFisher Scientific) per manufacturer’s instructions. Protein concentration was measured using the Pierce BCA-protein assay kit (ThermoFisher Scientific) per manufacturer’s instructions. Lysates were reduced, denatured and separated by SDS-PAGE, then transferred onto nitrocellulose membranes and blocked with 5% milk in TBS-T, then probed with primary and secondary antibodies of interest and by development using ECL (GE Healthcare). Band densitometry was measured using Image J software. Intensity was determined by subtracting background intensity compared to β-actin or Lamin B1.

### Quantitative Real Time RT-PCR

MDM monolayers were lysed with TRIzol (Invitrogen). RNA was isolated using chloroform extraction and ethanol precipitation then converted into cDNA using the ThermoScript RT-PCR system (Invitrogen). Quantitative PCR was performed using SYBR Green (Applied Biosystems). Genes of interest were normalized to GAPDH. Relative copy number (RCN) was determined using the formula: RCN = *2*^−ΔCt^ × 100, where ΔCt is the Ct_(target)_ − Ct_(reference)_. Fold change was calculated by comparing treatment groups to resting controls.

### Atomic absorption spectrometry

Donor serum diluted to concentrations of 0, 1, 2 10, 20 and 50% in RPMI 1640 or ZnSO_4_ diluted to concentrations of 0, 1, 10, 18 μM in RPMI 1640 containing 2% donor serum were assayed by atomic absorption spectrometry using AAnalyst 400 (PerkinElmer).

### C/EBPβ activity assay

Nuclear lysates were obtained from MDMs following treatment or control conditions as previously described using NE-PER nuclear and cytoplasmic extraction reagents purchased from Thermo Scientific (Rockford, IL) per manufacturer’s instructions. Nuclear localization of activated C/EBPβ in MDM nuclear lysates was then determined using the TransAM^TM^ C/EBPβ Transcription Factor Assay purchased from Active Motif (Carlsbad, CA) per manufacturer’s instructions. Absorbance at 450 nm was read by spectrophotometry.

### Confocal microscopy

MDMs (1.5 x 10^5^) were cultured on glass coverslips, then following 30 min, 24 h or 48 h treatment with or without LPS (100 ng/mL) and with or without ZnSO_4_ 18 μM, were washed three times with RPMI and exposed to Zinpyr-1 (Sigma-Aldrich) (1 μM) for 15 min. MDMs were then washed three times with DPBS, fixed with 4% paraformaldehyde for ten minutes, washed three times with DPBS and mounted to slides using ProLong Gold AntiFade Mounting media plus DAPI (Invitrogen Life Technologies). The slides were then examined by confocal microscopy using a FluoView 1000 Laser Scanning Confocal microscope (Olympus BX61). The mean fluorescence intensity (MFI) of random confocal images for approximately 150 macrophages per coverslip in duplicate for each experiment was quantified using pixel intensity measurement (NIH Image J program). MFI was calculated by dividing the green fluorescent signal above threshold by the number of DAPI stained nuclei for each image. Fold Zinc (MFI) was determined by averaging the MFI for each experimental group and dividing that value by the average resting control group MFI value. Fold change values were then averaged among independent experiments.

### Statistics

Each experiment was conducted a minimum of 3 times with different donors. Prism-5 software (Version 5.04; GraphPad) was used for linear regression analysis and to determine statistical significance between means. An unpaired, one-tailed Student’s t-test was used to analyze differences between two groups in most figures depicting cumulative data. A one-tailed Wilcoxon matched-pairs signed rank test was used to analyze differences between groups for cumulative data being compared to a baseline value. Significance was *p* < 0.05 for both tests and is depicted as * or #, respectively.

## Results and Discussion

### LPS induces ZIP8 in human macrophages

**Fig 1:**
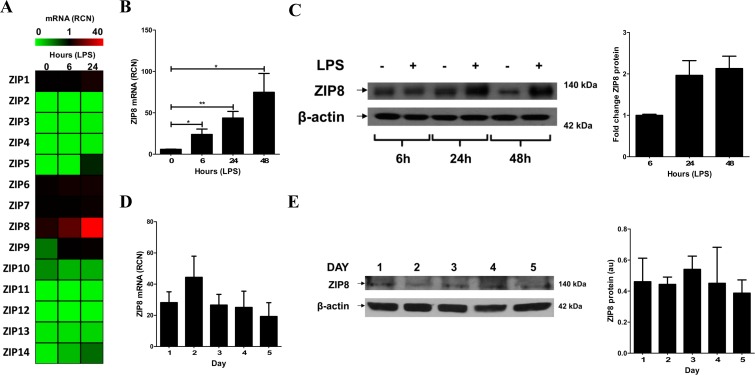
ZIP8 is constitutively present and highly inducible by LPS in human macrophages. **(A)** The mRNA expression profile of all ZIPs revealed that LPS (100 ng/mL) exposure significantly increases expression of ZIP8 in MDMs over time as determined by qRT-PCR. In contrast, the basal levels of the 13 other ZIPs were minimal and LPS exposure did not alter the expression of these ZIPs. **(B)** ZIP8 mRNA in MDMs is significantly induced following LPS (100 ng/mL) exposure. **(C)** ZIP8 protein levels in MDMs are significantly induced by LPS (100 ng/mL) exposure compared to resting unstimulated cells. Cumulative densitometric analysis reveals that ZIP8 protein levels increased two-fold by 24 h following LPS exposure. **(D, E)** ZIP8 mRNA and protein levels are maintained through monocyte to macrophage differentiation as determined by qRT-PCR and Western blot analysis. Cumulative densitometric analysis reveals that ZIP8 protein is constitutively present during differentiation. (A, B and D were quantified relative to GAPDH). Panels A, B and D are cumulative data from three different donors (mean ± SEM; *p < 0.05; **p < 0.01). Panels C and E are each representative of 3 experiments.

Import of extracellular zinc into the cytosol is achieved mainly through fourteen ZIP transporters which are differentially regulated and expressed in distinct cell types as a function of intracellular and extracellular cues [8]. In particular, ZIP8 is typically not highly expressed constitutively but is inducible by inflammation in monocytes [12]. Accordingly, we first examined the capacity of LPS to influence the expression of all ZIPs (1–14) in human macrophages via mRNA expression in MDMs cultured in RPMI containing 10% autologous serum from individual donors by qRT-PCR. We observed that LPS induces macrophage ZIP8 expression without significantly altering mRNA expression of the thirteen other ZIPs in MDMs (Fig 1A), and at an order of magnitude greater than the changes observed in any of the other ZIPs. ZIP8 was present in resting MDMs and ZIP8 mRNA was significantly induced following LPS exposure by over 10-fold, which led to induction of additional ZIP8 protein production in MDMs over time (Fig 1B and 1C). Consistent with previously published reports [27], ZIP8, an eight trans-membrane spanning protein, appeared as a heavily glycosylated isoform with an apparent molecular mass of approximately 140 kDa as determined by Western blotting. Following LPS exposure, macrophage ZIP8 mRNA levels increased by 6 h and peaked at 48 h while protein levels increased at 24 h and remained elevated through 48 h (Fig 1B and 1C). The relative equivalence of protein levels at 24 and 48 hours indicates that further elevation of ZIP8 mRNA beyond that observed at 24 hours may not result in additional protein production. During monocyte to macrophage differentiation, ZIP8 mRNA and protein were constitutively expressed, albeit at lower levels than what was observed following macrophage LPS stimulation (Fig 1D and 1E). These findings identified ZIP8 as the predominant LPS inducible importer of macrophage zinc. Furthermore, it became clear that in comparison to previous reports in monocytes [12], macrophages produce appreciable amounts of ZIP8 protein at rest, indicating that ZIP8 has the capacity to modulate intracellular zinc levels both within resting macrophages and to a greater extent following LPS exposure.

### Zinc modulates LPS-induced macrophage cytokine production

The inflammatory profile elicited by LPS-exposed macrophages is dependent in part upon micro-environmental zinc availability and ZIP8-mediated importation. The capability of extracellular zinc to impact cytokine production is predicated largely upon its activity as an intracellular second messenger following transport across the plasma membrane. Here we demonstrate that human macrophages exposed to LPS use extracellular zinc concentrations within the physiological range to modify inflammatory balance in the local milieu. The impact of zinc on LPS-induced macrophage cytokine production was investigated by supplementation of zinc sulfate into the media at different concentrations during LPS exposure. Analysis of increasing concentrations of donor serum in RPMI 1640 by atomic absorption spectroscopy predicted an average serum zinc concentration of approximately 68 ug/dL (S1A Fig), which is within the accepted normal range (66 to 110 ug/dL) [28]. Further, addition of 0, 10, 18 and 40 μM zinc sulfate into RPMI 1640 containing 2% autologous human serum, produced media zinc concentrations that were negligible (< 5 ug/dL), below (45 ug/dL), within (68 ug/dL) or above (147 ug/dL) normal, respectively (S1B Fig). MDMs were rested or exposed to LPS (100 ng/mL) and/or zinc sulfate for a period of 6 or 24 h.

**Fig 2:**
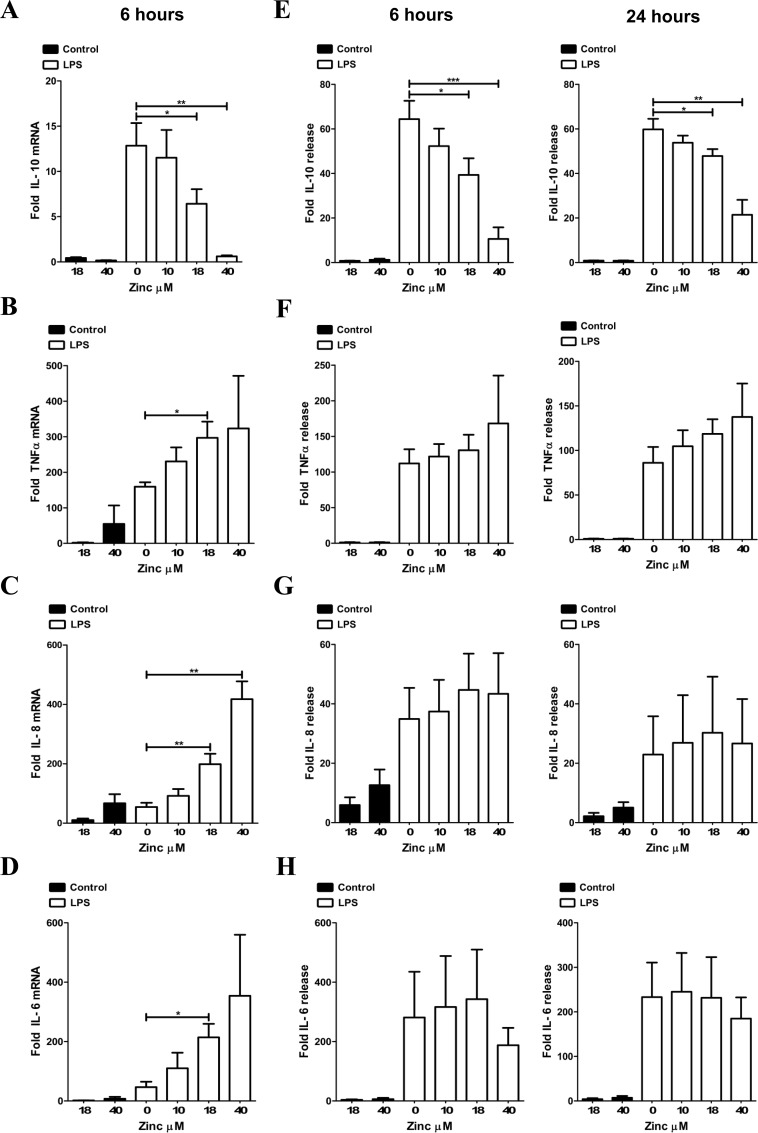
Zinc supplementation reduces macrophage IL-10 production in response to LPS. Zinc co-administration during LPS (100 ng/mL) stimulation of macrophages at 6 h decreases (**A**) IL-10 but increases (**B**) TNFα, (**C**) IL-8, and (**D**) IL-6 mRNA expression as determined by qRT-PCR (experimental groups quantified relative to GAPDH and normalized to resting controls). Zinc also decreases (**E**) IL-10 but trends toward an increase of (**F**) TNFα, (**G**) IL-8 and (**H**) IL-6 protein release at 6 h and 24 h as determined by ELISA (experimental groups normalized to resting controls). Panels A, B, C and D or E, F, G and H are cumulative data from 3 or 4 different donors, respectively (mean ± SEM; *p < 0.05; **p < 0.01; ***p < 0.001).

Zinc co-administration during LPS exposure significantly reduced IL-10 mRNA expression (Fig 2A) and protein release (Fig 2E). The impact of zinc on IL-10 release was highly reproducible among donors. In contrast, expression of TNFα, IL-8 and IL-6 mRNA (Fig 2B, 2C and 2D) and corresponding protein release (Fig 2F, 2G and 2H) were increased by co-administration of zinc with LPS. IL-10 production is increased in response to pro-inflammatory cytokines including TNFα [29]. Therefore zinc-induced increases in autocrine or paracrine signaling by TNFα, may in part account for the diminished inhibitory effect of zinc on IL-10 release between 6 and 24 h (Fig 2E and 2F). Previous studies using human monocyte cell models have shown that zinc can impact the extent of inflammation but results have varied likely due to differences in the approach utilized. Specifically, zinc supplementation during LPS exposure can both enhance and inhibit monocyte-derived pro-inflammatory cytokine release depending on the dose and timing of zinc exposure [30, 31]. Furthermore, both direct depletion and enrichment of intracellular zinc using the zinc chelator TPEN or the membrane permeable molecule pyrithione in concert with zinc, respectively, can reduce TNFα and IL-1β release following LPS exposure in monocytic cells [32]. To our knowledge, prior studies have not examined the extent to which zinc impacts immune function in primary human macrophages. Macrophage cytokine release controls the inflammatory balance within the local tissue environment. Synchronous production of both pro-inflammatory and immune modulatory cytokines including IL-10 regulates this balance. Our findings support the paradigm that macrophages utilize available zinc within distinct tissue compartments to increase local inflammation through elevation of TNFα, IL-8 and IL-6 while simultaneously reducing IL-10. In our model, we chose to examine zinc-mediated effects by recapitulating the physiologic zinc concentrations encountered in humans, thereby evaluating zinc-dependent changes in inflammation that occur *in situ*. It should be noted that zinc has been shown to directly bind LPS and increase potency in PBMCs [33]. However, in our macrophage model, zinc reduced IL-10 expression and release while simultaneously increasing that of other cytokines and chemokines thereby not supporting a generalizable increase in LPS potency.

### ZIP8 regulates macrophage IL-10 release

**Fig 3:**
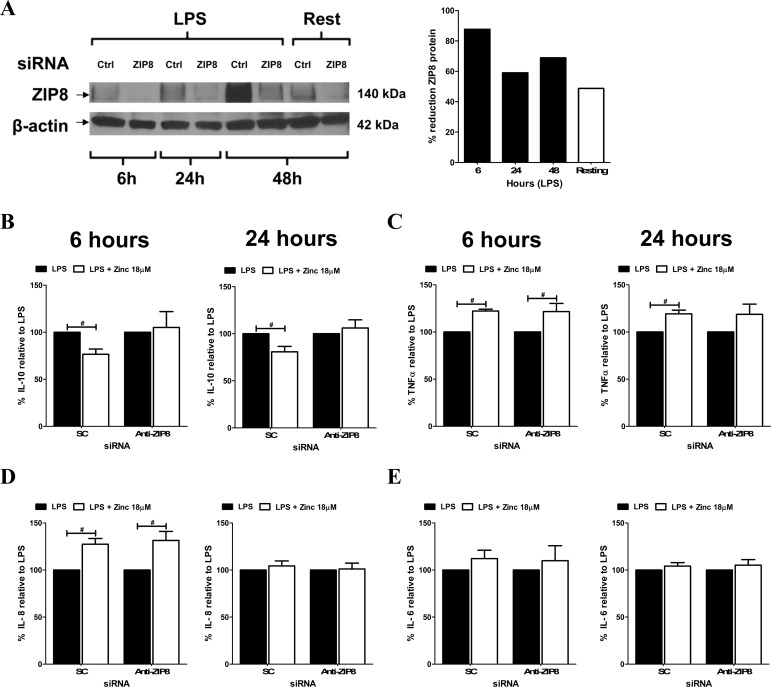
Reduction in macrophage IL-10 production by zinc is ZIP8-dependent. ZIP8 protein levels (**A**) are reduced following LPS (100 ng/mL) exposure in MDMs transfected with siRNA targeting ZIP8 (anti-ZIP8) compared to scramble control (SC) siRNA. Densitometric analysis of Panel 3A reveals that ZIP8 protein levels are reduced by over 60% following knockdown. Zinc-dependent reduction in macrophage (**B**) IL-10 production but not (C) TNFα (D) IL-8 and (E) IL-6 production is ZIP8-dependent as determined by ELISA of cell free supernatants following ZIP8 knockdown or scramble control and 6 or 24 h co-stimulation with LPS (100 ng/mL) and ZnSO_4_ (18 μM). Panel A is representative of 3 experiments. Panels B, C, D and E are cumulative data from 5 different donors (mean ± SEM; ^**#**^p < 0.05).

Given the constitutive and highly inducible expression of ZIP8 in macrophages, we next sought to determine whether ZIP8 was responsible for zinc-mediated cytokine changes in macrophages. First, ZIP8 was knocked down in MDMs by transfection with ZIP8 or scramble control siRNA. The impact of ZIP8 or control knockdown-treated MDMs were determined after 24 h exposure to LPS (100 ng/mL) with or without zinc sulfate (18 μM) supplementation. ZIP8 knockdown in MDMs was verified by Western blot analysis showing a consistent and durable knockdown of greater than 60% percent out to 48 h (Fig 3A). Importantly, analysis of the other 23 human zinc transporters by qRT-PCR revealed that only ZnT1 mRNA was significantly reduced as a consequence of ZIP8 knockdown following 24 hours of LPS exposure (S1 Table). ZnT1 expression decreases when cytosolic zinc levels decline [34]. Therefore, reduced ZnT1 expression during ZIP8 knockdown is consistent with reduced cytosolic zinc concentrations through loss of ZIP8-mediated zinc import. Loss of ZIP8 may also drive increased import by ZIPs or export by ZnTs without altering their mRNA or protein production by inducing redistribution or altering dimerization [8].

Cytokine analysis revealed that zinc-dependent inhibition of IL-10 release at 6 and 24 h following LPS exposure (Fig 3B) was reversed by ZIP8 knockdown, indicating that ZIP8 plays a vital role in the zinc-dependent reduction of macrophage IL-10. ZIP8 knockdown did not further alter zinc-induced increases in TNFα, IL-8 and IL-6 (Fig 3B, 3C and 3D) indicating ZIP8 specificity towards IL-10 regulation but not to the other factors that we examined. Importantly, we first observed zinc-dependent reductions in IL-10 at 6 h following LPS exposure (Fig 2A and 2E) and prior to LPS-induced increases in ZIP8 protein (Fig 1C). Taken together, these results indicate that the constitutively present pool of ZIP8 (Fig 1C and 1E) is sufficient to facilitate Zn-mediated reduction in the initial wave of IL-10 expression and that the induced pool of ZIP8 over time sustains this inhibitory effect, presumably for longer periods of IL-10 inhibition during prolonged pathogen LPS exposure.

Cumulatively, our findings indicate that mononuclear phagocytes have evolved to use ZIP8-dependent zinc to optimize the host inflammatory response to fit the context in which it is required. Zinc is unique relative to other micronutrients because of its multifaceted impact on cellular function. It is broadly essential for protein structure, catalytic function [35] and protection from oxidant damage [36] but is also utilized in specific signal transduction pathways as a tightly regulated second messenger [4, 37, 38]. Given that monocytes and macrophages are in different tissue compartments and produce distinct cytokine and chemokine profiles, it is to be expected that they use zinc to regulate inflammatory responses differently. Moreover, our findings suggest that innate immune host defense mediated by human macrophages is most likely dysregulated in the setting of zinc deficiency. In this context, IL-10 serves as a negative feedback regulator of TNFα, IL-6, IL-8, proIL-1β and IL-12 [15, 16, 23, 24, 39] production. Accordingly, we predict that significant alteration in macrophage IL-10 release in the setting of zinc deficiency during the initial response to pathogens has the potential to adversely impact both local and systemic inflammation and thereby alter the clinical course of infection.

### ZIP8 modulates macrophage zinc import in response to LPS

**Fig 4:**
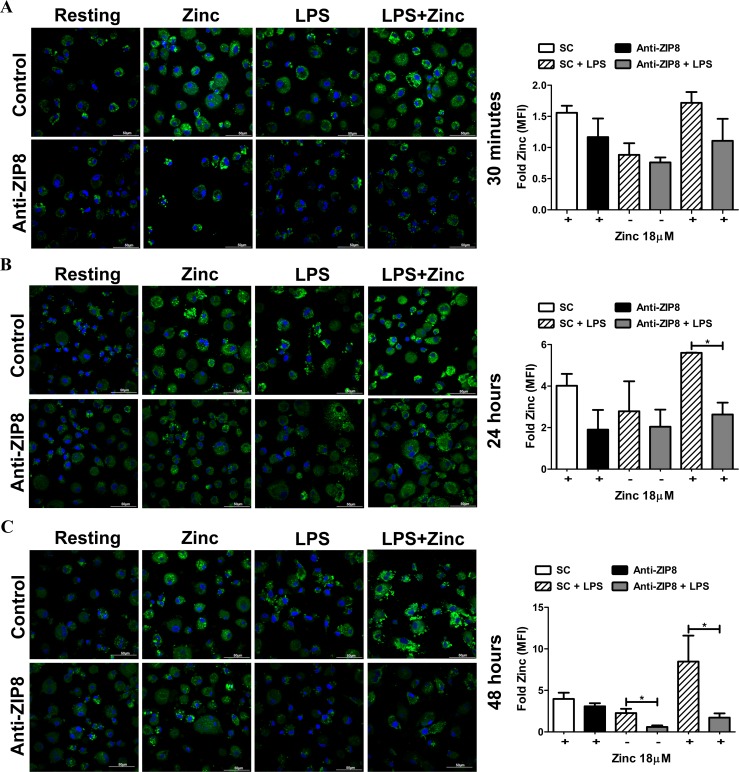
ZIP8 increases zinc accumulation in macrophages. Increases in macrophage intracellular zinc concentrations as determined by Zinpyr-1 staining in MDMs transfected with siRNA targeting ZIP8 (Anti-ZIP8) compared to scramble control (SC) siRNA after (**A**) 30 min (**B**), 24 hours and (**C**) 48 hours of zinc supplementation (18 μM) or zinc supplementation during LPS (100 ng/mL) exposure are reduced by ZIP8 knockdown compared to scramble control. Fold (MFI) was determined relative to resting control. Representative images and cumulative data from 3 different experiments conducted in duplicate (mean ± SEM; *p < 0.05). Scale bars, 50 μm.

ZIP8 resides on the plasma membrane and membrane of intracellular vesicles and organelles [4, 12, 27, 38, 40]. From these locations it can direct vesicular zinc efflux in T-cells [38] and increase intracellular zinc in monocytic cell lines [4, 12]. We previously determined that ZIP8 is localized to both the plasma membrane and intracellular vesicles in MDMs following LPS exposure [4]. Based on these findings and our observation that ZIP8 is responsible for reduction of LPS-induced IL-10 release by zinc supplementation, we next sought to determine whether ZIP8 increases macrophage uptake of zinc during LPS challenge. Following ZIP8 knockdown, macrophages were exposed to LPS (100 ng/mL) with or without zinc sulfate (18 μM) for 30 min, 24 or 48 h. Cells were stained with the zinc-specific fluorophore Zinpyr-1 prior to fixation with paraformaldehyde, then stained with DAPI and viewed by fluorescence confocal microscopy. Consistent with previously published reports [41], zinc localized within punctate inclusions within the cytosol. Zinc supplementation increased cellular zinc levels as early as 30 min after exposure (Fig 4A). Further increases in cytosolic zinc content occurred as a consequence of LPS exposure during Zn supplementation that began by 24 h (Fig 4B) and continued to increase up to 48 h (Fig 4C). Consistent with our hypothesis, ZIP8 knockdown resulted in a robust reduction in macrophage zinc accumulation at all time points. Significant differences were observed between ZIP8 knockdown and scramble control groups in LPS-exposed zinc-supplemented macrophages at 24 and 48 h (Fig 4A, 4B and 4C). These findings demonstrate that ZIP8 is responsible for zinc uptake within minutes as a result of constitutive ZIP expression in macrophages as well as over a sustained time frame consequent to induction of ZIP8 expression (Fig 1B and 1C)

In addition to zinc transport, ZIP8 participates in cytosolic influx of manganese, iron, cadmium and selenite. Iron and zinc inhibit the ZIP8-mediated uptake of one another [40, 42, 43]. Therefore, these cations have the potential to modulate the impact of zinc on macrophage immune function through competitive uptake and cell signaling [44, 45]. Although, Zinpyr-1 is highly specific for labile zinc it may be limited in its ability to distinguish between particular divalent cations under certain circumstances [46]. The cumulative impact of ZIP8 on macrophage inflammation in the presence of multiple substrates will require further study. Our findings obtained in macrophages are consistent with previous work that identified both “early” zinc-mediated signaling events (seconds to minutes) and “late” signaling events (minutes to hours), which are both critical for effective signal transmission during pathogen recognition [32, 47]. In this regard our data indicate that ZIP8 can play a role in both signaling phases in macrophages since it is constitutively present and highly inducible.

### Zinc supplementation modulates C/EBPβ nuclear localization and activity

Our previous work revealed that LPS induces the expression of ZIP8 through NF-κB [4]. The importation of zinc into the cytosol reduces subsequent nuclear localization of NF-κB subunit p65 through direct zinc binding and inhibition of the upstream IKK-complex [4]. However, these observations were restricted to human monocytes and cell lines. Our current findings reveal a distinct, ZIP8-dependent zinc effect on macrophage cytokine and chemokine production highlighted by a reduction in IL-10 release. In order to elucidate the underlying mechanisms responsible for that effect; we next evaluated the impact of zinc on the LPS-inducible kinases and transcription factors known to regulate macrophage IL-10 expression.

**Fig 5:**
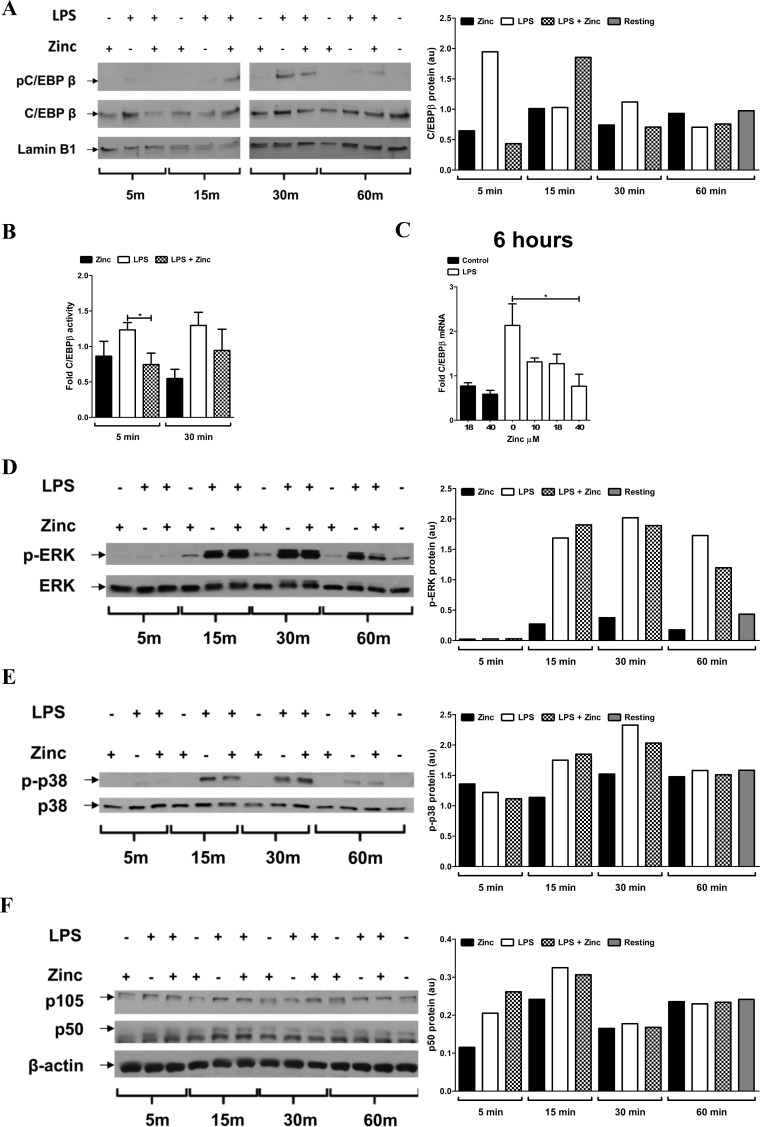
Zinc inhibits LPS-induced C/EBPβ. During LPS challenge zinc alters (**A**) C/EBPβ nuclear accumulation and phosphorylation beginning at 5 min and out to at least 30 min as determined by Western analysis of nuclear extracts and reduces both (**B**) C/EBPβ activity at 5 min as determined by TransAM ELISA of nuclear extracts and (**C**) transcription of C/EBPβ at 6h as determined by qRT-PCR. Zinc reduces (**D**) ERK phosphorylation at 60 min, but does not significantly alter (**E**) p38 phosphorylation (**F**) or p105/p50 degradation, as determined by Western analysis of whole cell lysates, following 5, 15, 30 or sixty min co-stimulation with LPS (100 ng/mL) and ZnSO_4_ (18 μM). Panels A, D, E and F are paired with densitometry and representative of 3 experiments. Panels B and C are cumulative data from 3 donors (mean ± SEM; *p < 0.05).

Accordingly, we determined the impact of zinc sulfate (18 μM) following LPS (100 ng/mL) exposure on the expression, phosphorylation, and activity of C/EBPβ in macrophages. We also determined the impact of zinc on the expression and extent of phosphorylation of the MAP kinases p38 and ERK and p50. Zinc supplementation during LPS exposure led to oscillation of C/EBPβ nuclear accumulation and phosphorylation that was consistently characterized by a rapid reduction of protein at 5 min, followed by an increase at 15 min, and again, a decrease at 30 min (Fig 5A). C/EBPβ nuclear activity was also reduced by the addition of zinc (18 μM) during LPS challenge at 5 min (Fig 5B). Additionally, mRNA levels of C/EBPβ were decreased by zinc supplementation following 6 h exposure to LPS (Fig 5C). Zinc supplementation also caused fluctuation in ERK phosphorylation with a consistent reduction in ERK phosphorylation at 60 min after LPS (Fig 5D). In contrast, zinc supplementation minimally impacted p-p38 (Fig 5E) and p50/p105 (Fig 5F) levels. Based on these observations, we propose that the import of extracellular zinc leads to a reduction in C/EBPβ-dependent and possibly ERK-dependent IL-10 transcription and protein production. To our knowledge this is the first time that zinc has been shown to reduce nuclear C/EBPβ protein content and activity.

C/EBPβ is activated following macrophage recognition of extracellular [48] or intracellular pathogens [49] and is indispensable for macrophage bacterial killing [50]. Consistent with our findings (Fig 5A, 5B and 5C), C/EBPβ is constitutively present in primary human macrophages [51, 52] and LPS increases C/EBPβ gene expression [51, 53] that corresponds with an increase in protein levels and DNA binding activity in murine macrophages [54, 55]. C/EBPβ-dependent transcription is essential for macrophage cytokine expression, including IL-10, in response to LPS [56–59]. Therefore, inhibition of C/EBPβ nuclear accumulation (Fig 5A) and activity (Fig 5B) by zinc occurs following zinc-dependent reduction of IL-10 expression and release consequent to LPS challenge. C/EBPβ is auto-regulated in response to LPS [60, 61], therefore the observation that zinc reduces LPS-induced C/EBPβ mRNA levels (Fig 5C) demonstrates that zinc is able to reduce its transcriptional activity. Accordingly, reduced C/EBPβ mRNA levels contribute to reduction of C/EBPβ-driven IL-10 production. MAPK signaling is also necessary for LPS-dependent IL-10 induction through C/EBPβ [21]. In murine macrophages, C/EBPβ transcription, DNA binding [55] and cytokine production [62–64] is induced through p-38 kinase. In our model, zinc-mediated inhibition of C/EBPβ accumulation and phosphorylation in human macrophages did not decrease p38 phosphorylation (Fig 5E), which indicates that zinc modulates the activity of a yet to be identified kinase(s) or phosphatase(s) upstream of C/EBPβ in a TLR4-dependent signaling pathway. ERK is also important to macrophage pro-inflammatory cytokine [65] and IL-10 production [17, 19, 20]. Consistent with our findings, zinc has been shown to inhibit ERK phosphorylation in airway smooth muscle cells [66]. Myeloid cell IL-10 production is proportional to the level of ERK activation [67]. Specifically, LPS induces ERK phosphorylation through activation of the IKK complex in the canonical NF-κB pathway. However, in murine macrophages LPS-induced DNA binding of C/EBPβ, which is reduced by MAPK inhibition is independent of NF-κB signaling [21] and overexpression of C/EBPβ, did not increase p50-mediated IL-10 transcription [18]. Our model supports a zinc-mediated, p50-independent induction of IL-10 by C/EBPβ following LPS exposure (Fig 5A and 5D) that is regulated through ZIP8.

Although pro-inflammatory cytokines including TNFα and IL-6 are induced by C/EBPβ, their mRNA levels did not decrease following zinc-dependent reduction of C/EBPβ (Figs 2B, 2C, 2D, 5A and 5B). C/EBPβ availability in part determines its activity [11], such that zinc-dependent alteration of its abundance may alter cytokine expression differentially.

Increases in microenvironmental tonicity induce further inflammation in LPS-stimulated macrophages [68, 69]. Osmotic stress generated from our addition of zinc sulfate may further help to explain elevations in pro-inflammatory cytokines. Regulation of macrophage cytokine production is complex and regulated at many different levels. It is likely that the impact of zinc in general and ZIP8-dependent zinc is multifactorial. Post-transcriptional regulation by microRNAs modulate expression of ZIP transporters including ZIP8 [70, 71] as well as production of pro-inflammatory cytokines [72] and IL-10 [73]. MicroRNA expression is also subject to regulation by zinc [74, 75]. Further investigation of zinc-dependent modulation of cytokine signaling in human macrophages is essential to understanding the mechanisms underlying our observations.

In summary, we show that extracellular zinc reduces human macrophage IL-10 production following LPS exposure. Most striking, the zinc-dependent effect on IL-10 is facilitated in large part by the zinc transporter ZIP8, which is both constitutively expressed and further induced by LPS. ZIP8 increases macrophage zinc uptake and accumulation. Zinc bio-redistribution into the cytosol leads to an immediate alteration in C/EBPβ nuclear accumulation and activity as well as a reduction in ERK phosphorylation through mechanisms that remain to be fully defined. Based on our findings, we speculate that ZIP8-mediated reduction of IL-10 and increase in TNFα, IL-6, and IL-8 by tissue macrophages may serve to facilitate more rapid resolution of infection and limit bacterial dissemination and further, that this regulation requires physiologic amounts of zinc within the local milieu. We believe that the fundamental observations made herein have important implications for innate immune defense, particularly against intracellular bacterial pathogens that manipulate IL-10 production and also underscore the importance of proper zinc nutrition to optimize protective immune responses to infection.

## Supporting Information

S1 Fig Modeling serum zinc levels in RPMI media. (A) Levels of zinc in human donor serum diluted in RPMI at different concentrations or (B) zinc sulfate diluted at different concentrations in RPMI containing 2% donor serum were determined by Atomic Absorption Spectrometry and equations were generated by regression analysis for prediction of zinc levels within the physiological range. Panels A and B are cumulative data from 3 different donors (mean ± SEM).
(TIF)

S1 Table The impact of ZIP8 knockdown on macrophage ZIP and ZnT expression. The mRNA expression profile of all ZIPs and ZnTs after ZIP8 knockdown or treatment with a scrambled control followed by LPS (100 ng/mL for 6, 24 or 48 h) exposure reveals that reduction in ZIP8 is the only major change in ZIP expression and reduction of ZnT1 at 24 h after LPS is the only major change in ZnT expression following ZIP8 knockdown as determined by qRT-PCR relative to GAPDH. S1 Table represents cumulative data from 3 different donors (mean values).
(XLSX)
