# Supplementary figures and images for: Zinc Modulates Endotoxin-Induced Human Macrophage Inflammation through ZIP8 Induction and C/EBPβ Inhibition
Source: PLoS One. 2017 Jan 5;12(1):e0169531. doi: 10.1371/journal.pone.0169531 (PMC5215883; doi:10.1371/journal.pone.0169531)

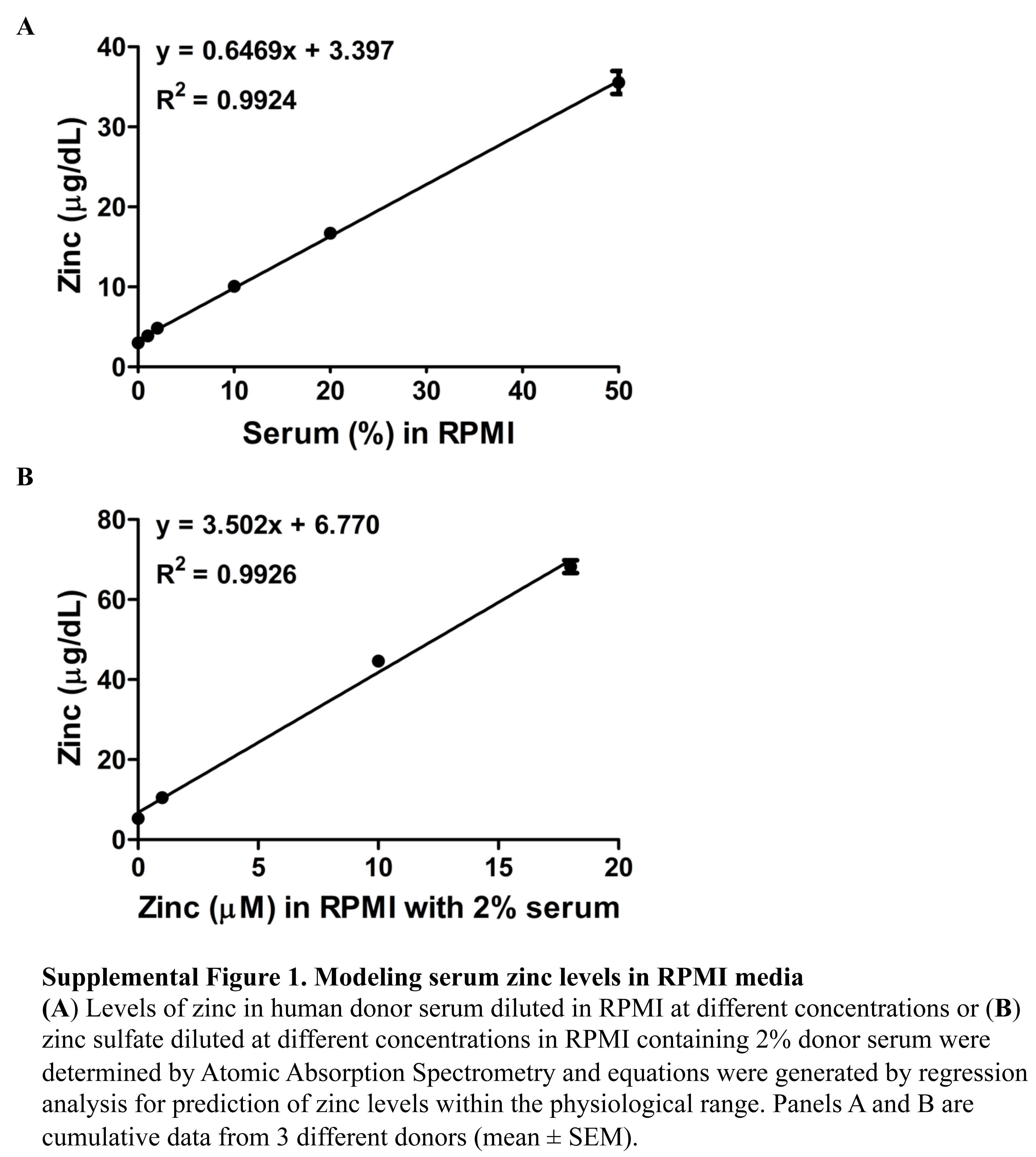

Supplement: S1 Fig — (A) Levels of zinc in human donor serum diluted in RPMI at different concentrations or (B) zinc sulfate diluted at different concentrations in RPMI containing 2% donor serum were determined by Atomic Absorption Spectrometry and equations were generated by regression analysis for prediction of zinc levels within the physiological range. Panels A and B are cumulative data from 3 different donors (mean ± SEM). (TIF) [file pone.0169531.s001.tif]
